# Rate of fibular non-union in patients with tibial shaft fractures: a retrospective cohort study

**DOI:** 10.1007/s00590-026-04711-3

**Published:** 2026-05-22

**Authors:** W. Andrew Day, C. Julian Clark, Johnathan W. Riley, Drew P. Melancon, Isaac J. Spears, J. C. Davidson, Lauren Hopper, R. Peyton Parker, Priyanka Nehete, Peter N. Mittwede, Patrick F. Bergin

**Affiliations:** 1 University of Mississippi Medical Center, Jackson, USA; 2 Department of Orthopaedic Surgery, University of Tennessee Health Science Center, Chattanooga, USA

**Keywords:** Fibular non-union, Tibial shaft fracture, Intramedullary nailing, Fracture fixation, Intramedullary flexible nail (IMFN), Plate fixation, Radiographic union (RUST/mRUST), Fracture location, Orthopaedic trauma, Retrospective cohort study

## Abstract

**Purpose:**

Fibular non-union occurs at an unknown rate in patients with tibial shaft fractures. The purpose of this study was to assess the rate of fibular non-union in patients with tibial shaft fractures. We also assessed if any factors are associated with the rate of fibular non-union.

**Methods:**

This was an Institutional Review Board (IRB) approved retrospective cohort study performed at a level one trauma center. We reviewed the collected data on 400 patients that had fibular fractures with associated tibial shaft fractures from June 2012 to June 2021. To meet the inclusion criteria for this study, patients 16 years and older needed a minimum of three months of follow-up and sustained fibular fractures with associated tibial shaft fractures fix with an intramedullary rod. The standard and modified radiographic union scale in tibia (RUST and mRUST) fractures score of 8 was used as the parameter to determine fibular union.

**Results:**

The rate of fibular non-union was 21% (84/400). Distal 1/3 fibular fractures had a higher non-union rate (25%, 46/179) than proximal and middle 1/3 fractures (12%, 10/83; 20%, 28/138 respectively) (*p* = 0.036). Patients that had fibular fixation went on to form a non-union at a rate of 25% (31/121) and patients that did not have fibular fixation went on to form non-union at a rate of 19% (52/279) (*p* = 0.114). Of the patients that did have fibular fixation, those fixed with intramedullary flexible nail (IMFN) or screw are less likely to form a non-union than those fixed with plates (*p* = 0.049).

**Conclusion:**

This study identified the rate of fibular non-union in patients with associated tibial shaft fractures treated with an intramedullary rod. We also identified that there is an increased risk of non-union when there is fibular fixation with plates and screws versus IMFN. We do not know the clinical implications of this, but it may be a source of pain in patients with healed tibia shaft fractures therefore we believe a prospective randomized controlled study may be warranted.

**Supplementary Information:**

The online version contains supplementary material available at 10.1007/s00590-026-04711-3.

## Introduction

Fibular fractures commonly occur alongside fractures of the tibia or as a component of malleolar fractures in the ankle. In fact, up to 85% of tibia fractures are accompanied by a concomitant fibular fracture [1]. The fibula is typically considered accessory to the tibia as it only shares 6.4% of the weight distribution with the ankle joint in the neutral position; therefore, it is not always necessary to surgically repair the fibula [2]. Many surgeons are of the school of thought that most fibular fractures heal on their own without intervention. However, some do not heal, and can result in a painful, symptomatic non-union [3]. These painful sequelae negatively impact patients’ quality of life, and potentially lead to more surgery and the increased likelihood of infection and other complications [3]. 

Although recent evidence suggests that the rate of fibular non-union is increasing due to the use of interlocking intramedullary nailing of the tibia, there is a lack of information on delayed fibular union or non-union of the fibula in conjunction with a tibial fracture [3]. Rates of fibular non-union are low with studies citing ranges from 0% to as high as 29.4% with an average of 3.8% ± 5.8% [1, 3–10]. With rates this varied and multiple acceptable methods of management, it remains unclear what, if any, significance fibular non-unions have in the management of patients with lower extremity traumatic injuries. Initial management of fibular fractures can be conservative or operative. Operative modalities include open reduction and internal fixation/instrumentation via plates and screws, intramedullary nails, or intramedullary screws. Each of these different routes of management has their respective benefits and risks. It is still unclear, however, if any of these methods result in lower non-union rates. The current body of literature further obscures the matter as some studies show increased non-union rates with conservative [5] management and some show increased non-union rates with operative [11] management.

There is limited data available for fibular non-union with associated tibial shaft fracture. This retrospective cohort study assesses the rate of fibular non-union in patients with concomitant tibial shaft fractures and examines whether fibular fracture location, tibial union status, and fibular fixation method are associated with fibular non-union.

## Methods

A retrospective chart review of patients aged 16 years and older who sustained tibial shaft fractures with concomitant fibular fractures treated operatively from June 1, 2012, to June 14, 2021, was conducted using EPIC Hyperspace at our institution. Patients were excluded if they: (1) were younger than 16 years, as these patients are not managed by the orthopaedic trauma service; (2) presented initially with radiographically established non-union, delayed union, or infection, as these conditions would confound the primary outcome; or (3) had less than 90 days of radiographic follow-up. Our institution serves a large catchment area with a patient population that faces significant barriers to healthcare access, including limited transportation and economic disadvantage. Consequently, inconsistent follow-up is common in our practice setting, and the 90-day minimum follow-up criterion was selected to balance the need for adequate healing assessment while maintaining a representative sample of our trauma population.

The primary objective of the study was to determine the rate of fibular non-union in our cohort. Secondary objectives were determining the rate of fibular non-union when the fibula was instrumented vs. not, the rate of fibular non-union in association with BMI, tobacco use, diabetes, HbA1c, fracture morphology, fracture location along the fibula, and open vs. closed status. Clinical outcomes were assessed using radiographic outcomes only, time to non-union using Rust and modified rust criteria, rate of fibular non-union, and the impact of fibular stabilization on the rate of non-union. The radiographic union score for tibial (RUST) fractures and modified RUST (mRUST) are criteria that assess the anterior, posterior, medial and lateral cortices callus formation and fracture line visibility [13, 14]. The RUST criteria provides a score between 1 and 3 and mRUST provides a score between 1 and 4. A commonly accepted definition of a delayed union is failure to reach signs of radiographic union by 3 months. Thus, we calculated the rate of fibular non-union at 3 months follow up to determine the incidence.

All patients were treated under the orthopaedic department’s standard operating procedure for their presenting injury All tibias that underwent intramedullary nailing received nails from the same company using the same approach (suprapatellar), instrumented fibulas received either 1/3 tubular plates, intramedullary flexible nails or screw from the same manufacturer. All surgeries were performed by fellowship-trained orthopaedic trauma surgeons. Moreover, every patient received Cefazolin within 30 min of incision unless they had a penicillin allergy. In those instances, they received clindamycin. All nails were statically and the diameters ranged from 8 to 13. SPSS statistics was used for all data analysis (IBM Corp 2023 Vers. 29.0.2.0 Armonk, NY: IBM Corp). Fisher’s Exact Test or Chi-squared test was used for categorical data, and T-test was used for continuous data. Significance was set at < 0.05.

## Results

Our chart review returned 850 records meeting inclusion criteria specifically with the CPT code 27,759. Of those records, 155 did not have fibula fractures. Furthermore, 1 patient was under 16 years old, and 294 patients did not have adequate follow up: leaving us with a total of 400 records for inclusion. Of the 400 included patients, there were 252 males and 148 females, the mean age at time of injury was 40.1, and the mean BMI was 29.1 (further demographic details can be found in the Online Appendix).

The rate of fibular non-union for this cohort was 21% with 84 patients having radiographic non-union at 3 months. The non-union diagnosis was validated using the mRUST score, which assigns a score to the anterior, posterior, medial, and lateral cortices on radiograph and assigns a score from 1 to 4 based on callous formation and fracture line visibility, with scores totaling 8 or less being classified as non-union (*P* < 0.001). None of the demographic variables had any significant association with fibular non-union (Online Appendix). Fibular fracture morphology, open vs. closed fractures, and the presence of fibular fixation did not have any significant association with fibular non-union (Table 1). However, the location of the fracture along the fibula was significantly associated with non-union. In our cohort, distal third fractures (46/179, 25%)were more likely to progress to non-union than fractures in both the middle (28/138, 20%) and proximal third (10/83, 12%) (*P* = 0.036).

The method of fibular fixation did show that plates were significantly more likely to progress to non-union than flexible nail or intramedullary (IM) screw fixation (*P* = 0.049; Table 1). In sub-analysis of the fibulas that were instrumented, 34/121 had returns to the operating room (RTOR): 12 for infection and 22 for other reasons (coverage by plastic surgery, painful hardware, etc.). Of those that underwent hardware removal 12 were due to infection, 4 were due to non-union without infection, and 6 were for other reasons (Table 2).

Fibular non-union was significantly associated with the development of tibial non-union (*P* = 0.005, OR 1.28–4.58, Table 3). Tibial non-union was not significantly associated with the type of fibular fixation (*P* = 0.690). Fixation of the fibula was not associated with tibial non-union (*P* = 0.207), neither was the location of the fibula fracture (*P* = 0.377).

## Discussion

Our primary aim in this study was to determine the incidence of fibular non-unions in concomitant tibia fractures treated with intramedullary nail. Several factors may explain why our fibular nonunion rate of 21% is substantially higher than some previously reported rates (0–5.4%) but comparable to Lee et al.‘s 27.8% in their non-fixation group. First, despite similar surgical techniques—both studies utilized the Expert Tibia Nail (Synthes, Solothurn, Switzerland), though our institution employs a suprapatellar approach versus Lee et al.‘s lateral parapatellar or trans-patellar approach—our patient demographics differ considerably. Our cohort was younger (mean age 40 vs. 48.6 years), had higher BMI (29.1 vs. 25), broader age range (16–85 vs. 33–64 years), and different ethnic composition (predominantly African American vs. Korean population). These demographic differences may influence healing capacity and fracture outcomes. Second, injury severity and fixation patterns likely vary across studies. Our level one trauma center treats a high proportion of high-energy mechanisms and complex injuries. Additionally, 30% of our fibulas were instrumented compared to different fixation rates in other series, reflecting varying institutional practices and injury characteristics that complicate direct comparisons. Third, our strict radiographic definition of nonunion (mRUST score ≤ 8 at minimum 3 months follow-up) may capture delayed unions that other studies with longer follow-up periods (6–12 months) would classify as eventual union. This methodological difference in defining the outcome could substantially explain our higher reported rate. Finally, our patient population faces significant barriers accessing healthcare, including transportation limitations and insufficient financial means, and decreased health literacy which may result in delayed or inconsistent care impacting healing outcomes. These contextual factors must be considered when assessing the generalizability of our findings to other practice settings.

Non-union of the fibula is becoming increasingly more common in association with intramedullary nailing of concomitant tibial shaft fractures [3]. The current standard of managing tibial shaft fractures with IMN creates a dilemma for surgeons in regards to managing the fibula; which is injured in 80% of these cases [3]. The relative stability construct of an IMN relies on micromotion across the fracture site to facilitate healing of the bone. The increased distance of the fibula from these points of stability magnifies that motion leading to inappropriate amounts of strain that can lead to non-union [16]. On the contrary, stabilizing the fibula can add too much rigidity to the tibia construct and limit the needed micromotion and potentially delay healing [17]. 

There is a gap in the literature regarding the rates of non-union in relation to the type of fixation the fibula receives. We found that the rates of non-union were significantly higher in the plate and screw cohort (29%). To contrast, the incidence of non-union in the cohort whose fibulas were stabilized with a single intramedullary flexible nail (IMFN) was 15%. There were only two fibulas treated with intramedullary screws, and both went on to non-union; however, this sample is too small to make any meaningful conclusions.

One possible explanation for this difference is the difference in construct types and mechanisms of healing. The tibia is fixed using an intramedullary nail which confers relative stability and leads to secondary bone healing [18]. In the cohort fixed using an IMFN, this construct mirrors that of the tibia exposing the fracture to stress comparable to a tibia under normal physiologic conditions while maintaining similar stability. The plate cohort had two different modes of fracture healing where the tibia maintained relative stability and underwent secondary bone healing. On the other hand, plate fixation of the fibula creates absolute stability in non-comminuted fractures and provides a more rigid construct relative to the tibia in comminuted and segmental fibula fractures [18]. The increased stiffness reduces micromotion at the fracture site and acts as a stress shield on the fibula while maintaining the fracture gap [19]. The location and orientation of the plate typically involves the length of the plate running from the proximal end of the fracture to the distal portion, and the width running from the anterior cortex of the fibular to the posterior cortex (Table 1). This limits the axial stress across the fracture site specifically, whereas the IMFN allows for axial stress, which has been shown to promote bone healing (Table 2) [19].

Typically, the fibula is regarded as a model bone for fracture healing as it has a generous soft tissue envelope, endosteal blood supply, and relatively low exposure to stress [20]. However, there is less soft tissue and more stress placed across the fracture site towards the distal end of the fibula [5]. These factors contribute to the findings noted in prior investigations that the majority of fibula non-unions occur in the distal third [3, 5]. Our study corroborates these findings with distal third fibula non-unions occurring significantly more than at the middle or proximal third. Our study corroborated the findings that fibula non-unions are significantly associated with tibial non-unions as described by Ko and Lee [4]. 

This study has several limitations. First and foremost, retrospective studies are inherently prone to selection and information bias, with incomplete chart data and inability to control for unmeasured confounders such as nutritional status, weight-bearing compliance, or rehabilitation protocols. Second, as a single-center study at a level one trauma center serving a population with significant barriers to healthcare access, our findings may not be generalizable to other practice settings. Our patient population experiences limited transportation and economic disadvantage, resulting in inconsistent follow-up patterns. The 90-day minimum follow-up requirement may have excluded patients who healed uneventfully, and this may not accurately reflect the true non-union rate in our cohort. Third, while our cohort of 400 patients is substantial, subgroup analyses were limited by small sample sizes—particularly the intramedullary screw group (*n* = 2)—which may have reduced statistical power to detect clinically meaningful differences. Fourth, we did not systematically assess patient-reported outcomes, functional scores, or pain levels, limiting our understanding of the clinical significance of fibular nonunion. Finally, as an observational study without randomization, the choice of fixation method was based on surgeon judgment and injury characteristics, introducing potential confounding by indication that limits causal inference.

## Conclusion

To our knowledge, this is the first investigation into the incidence of fibular non-unions both with a cohort of this scale and with an analysis into the relationship between fibular non-unions and the various methods of open reduction and internal fixation used by orthopaedic surgeons. While our incidence of non-union was high, it appears to fall within the ranges of the literature referenced during our investigation. Our study demonstrates no significant difference in non-union rates between instrumented and non-instrumented fibulas; however, the significant reduction in non-union with the use of IMFN compared to plate and screws pushes us to consider whether it (IMFN) is the superior method of fixation in the event that a surgeon elects to fix the fibula. Future studies should be centered around prospective trials that directly compare fibular treatment strategies (observation alone versus IMFN versus plate fixation) in patients with tibial shaft fractures undergoing intramedullary nailing, with particular attention to non-union rates and functional outcomes, to establish evidence-based treatment algorithms.

**Table 1:** Fibular non-union rates stratified by fibular fracture characteristics and treatment

Injury characteristics	Total, *N*(%)	Non-union, *N*(%)	Union, *N*(%)	*P*-value (OR, CI)
*Fibular non-union*				
Yes	84 (21%)			
No	316 (79%)			
mRUST				< 0.001
*Fracture morphology*				
Segmental	56 (14%)	15 (27%)	41 (73%)	0.256
Comminuted	223 (56%)	48 (22%)	175(78%)	
Oblique	64 (16%)	15 (23%)	49 (77%)	
Transverse	35 (9%)	3 (9%)	32 (91%)	
Spiral	22 (5%)	3 (14%)	19 (86%)	
*Fracture location*				
Proximal 1/3	83 (21%)	10 (12%)	73 (88%)	0.036
Middle 1/3	138 (34%)	28 (20%)	110 (80%)	
Distal 1/3	179 (45%)	46 (25%)	133 (75%)	
*Open vs. closed*				
Open	212 (53%)	48 (23%)	164 (77%)	0.377 (1.24, 0.77–2.02)
Closed	188 (47%)	36 (19%)	152 (81%)	
*Fibular fixation*				
Yes	121 (30%)	31 (25%)	90 (75%)	0.114 (1.5, 0.9–2.5)
No	279 (70%)	52 (19%)	276 (81%)	
*Fixation type (% of fixation cohort)*				
Plate	65 (54%)	19 (29%)	46 (71%)	0.049
IM nail or screw	56 (46%)	8 (14%)	48 (86%)	

mRUST, modified radiographic union scale for tibias; OR, odds ratio; CI, 95%confidence interval

**Table 2:** Sub-analysis of non-union rates in instrumented fibulas

Injury characteristics		Plate, *N* (NU)	Nail, *N* (NU)	Screw, *N* (NU)
Total	121	65 (19)	54 (8)	2 (2)
Fracture location	Proximal 1/3	6 (1)	3 (0)	0
	Middle 1/3	13 (4)	13 (0)	1 (1)
	Distal 1/3	46 (14)	38 (8)	1 (1)
RTOR	34	19 (5)	13 (4)	2 (2)
	Infection	7 (3)	5 (3)	0 (0)
	Other	12 (2)	8 (1)	2 (2)
Hardware removal	22	12 (5)	8 (4)	2 (2)
	Infection	7 (3)	5 (3)	0 (0)
	Other	5 (2)	3 (0)	2 (2)

N, number; NU, non-union; RTOR, return to operating room

**Table 3:** Tibial non-union rates stratified by fibular fracture characteristics and treatment

Injury characteristics	Total, *N*(%)	Non-union, *N*(%)	Union, *N*(%)	*P*-value (OR, CI)
*Tibial non-union*				
Yes	50 (12.5%)			
No	350 (87.5%)			
mRUST				< 0.001
*Fibular non-union present*				
Yes	84(21%)	18 (21%)	66 (79%)	0.005 (2.42, 1.28–4.58)
No	316 (79%)	32 (10%)	284(90%)	
*Fracture location*				
Proximal 1/3	83 (21%)	11(13%)	72 (87%)	0.377
Middle 1/3	138 (34%)	21(15%)	117 (85%)	
Distal 1/3	179 (45%)	18(10%)	161 (90%)	
*Fibular fixation*				
Yes	121 (30%)	19 (16%)	102 (84%)	0.207 (1.48, 0.8–2.75)
No	279 (70%)	31 (11%)	248 (89%)	

mRUST, modified radiographic union scale for tibias; OR, odds ratio; CI, 95%confidence interval

## Supplementary Information

Below is the link to the electronic supplementary material.

Supplementary Material 1

Supplementary Material 2

## Data Availability

All raw data used for analysis is provided in the supplementary material in a standard, anonymous form.
