# Supplementary figures and images for: Rate of fibular non-union in patients with tibial shaft fractures: a retrospective cohort study
Source: Eur J Orthop Surg Traumatol. 2026 May 22;36(1):199. doi: 10.1007/s00590-026-04711-3 (PMC13197365; doi:10.1007/s00590-026-04711-3)

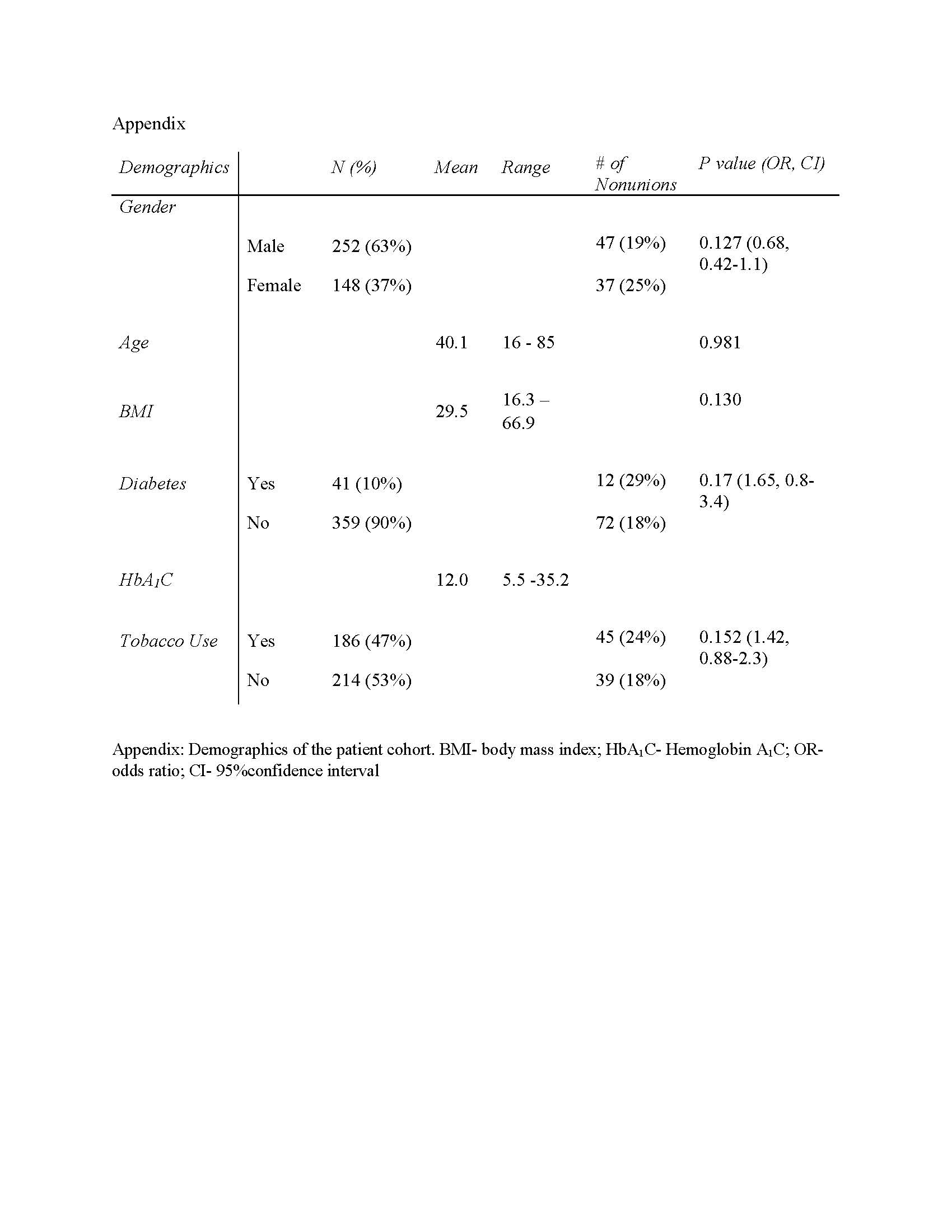

Supplement: Supplementary file 1 — Supplementary Material 1 [file 590_2026_4711_MOESM1_ESM.jpg]
